# Our Collaborators

**Published:** 1882-02

**Authors:** 


### EDITORIAL DEPARTMENT.

OUR COLLABORATORS.—We take this occasion to express our grateful appreciation to those gentlemen who have given us a promise to furnish original communications and clinical reports during the year, for the INDEPENDENT PRACTITIONER. The aid and encouragement thus offered has enabled us to make very great improvements in the contents and appearance of the journal. As said heretofore, we do not intend to rest satisfied until our readers agree with us that the perfection of medical journalism is reached.

We trust that our friends will send in their subscriptions at once and induce all their friends to subscribe also.

